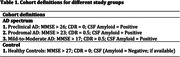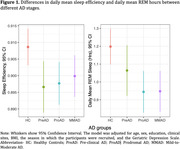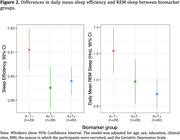# Associations of sleep efficiency and rapid eye movement sleep in early Alzheimer's disease (AD): Results of the RADAR‐AD study

**DOI:** 10.1002/alz.087259

**Published:** 2025-01-09

**Authors:** Alankar Atreya, Pauline Conde, Aiden Doherty, Srinivasan Vairavan, Marijn Muurling, Casper de Boer, Jelena Curcic, Margarita Grammatikopoulou, Spiros Nikolopoulos, Anna‐Katharine Brem, Neva Coello, Holger Fröhlich, Vaibhav Narayan, Gayle Wittenberg, Dag Aarsland, Cornelia M Van Duijn, Chris Hinds, the RADAR‐AD Consortium

**Affiliations:** ^1^ University of Oxford, Oxford United Kingdom; ^2^ King's College London, London United Kingdom; ^3^ Janssen Research and Development, Titusville, NJ USA; ^4^ Janssen Research and Development LLC, Titusville, NJ USA; ^5^ Alzheimer Center Amsterdam, Neurology, Vrije Universiteit Amsterdam, Amsterdam UMC location VUmc, Amsterdam Netherlands; ^6^ Novartis Institutes for BioMedical Research, Basel Switzerland; ^7^ Centre for Research & Technology Hellas, Thessaloniki Greece; ^8^ University Hospital of Old Age Psychiatry, University of Bern, Bern Switzerland; ^9^ King’s College London, London United Kingdom; ^10^ Fraunhofer Institute for Algorithms and Scientific Computing SCAI, Sankt Augustin Germany; ^11^ Davos Alzheimers Collaborative, Wayne, PA USA; ^12^ Johnson & Johnson Innovative Medicine, Titusville, NJ USA; ^13^ UK Dementia Research Institute, Institute of Psychiatry, Psychology & Neuroscience, King's College London., London United Kingdom; ^14^ Centre for Age‐Related Medicine, Stavanger University Hospital, Stavanger, Stavanger Norway

## Abstract

**Background:**

The relationship between sleep and AD is unclear: sleep problems may contribute to AD pathogenesis, but the spreading of AD pathology across the brain may also de‐regulate sleep. What aspect of sleep is relevant in which disease phase is also unclear, as many studies are based on questionnaires. We study sleep efficiency and rapid eye movement (REM) sleep, objectively measured using an activity tracker to shed light on sleep disturbances across the AD spectrum.

**Method:**

The RADAR‐AD is a cross‐sectional study (N=204), including healthy controls (HC, n=67), preclinical AD (preAD, n=34), prodromal AD (proAD, n=56) and mild‐to‐moderate AD (MMAD, n=47) participants (Table 1). Participants wore a Fitbit Charge‐3 activity tracker on their non‐dominant wrists for eight weeks, measuring REM sleep (in hours) and sleep efficiency (sleep duration/total time in bed). CSF amyloid and phosphorylated Tau (pTau) levels were determined in the subset (N=117) and stratified into biomarker groups (A‐T‐, A+T‐, and A+T+). We evaluated the difference in these mean sleep features among AD stages and biomarker groups.

**Result:**

Compared to HC, we observed reductions in daily mean REM sleep for proAD (Estimate±SE=‐0.24±0.08, p=0.002) and MMAD (Estimate±SE=‐0.23±0.08, p=0.001) (Figure 1), and in sleep efficiency for preAD (Estimate±SE=‐0.012±0.005, p=0.016), proAD (Estimate±SE=‐0.011±0.004, p=0.015), and MMAD (Estimate±SE=‐0.009±0.004, p=0.046) participants. Compared to A‐T‐ participants, sleep efficiency was reduced for A+T‐ (Estimate±SE=‐0.016±0.006, p=0.022) and A+T+ (Estimate±SE=‐0.013±0.006, p=0.019) participants (Figure 2), while REM sleep was lower for A+T+ participants only (Estimate±SE=‐0.32±0.11, p=0.004).

**Conclusion:**

Sleep efficiency is altered across the AD spectrum, including the early pre‐clinical AD stage. There is a trend toward lower REM sleep among subjects at more advanced stages of AD and elevated amyloid and tau that may suggest an association between AD pathology and REM sleep.

This work has received support from the EU/EFPIA Innovative Medicines Initiative Joint Undertaking (grant No 806999). www.imi.europa.eu. This communication reflects the views of the RADAR‐AD consortium, and neither IMI nor the European Union and EFPIA are liable for any use that may be made of the information contained herein.